# Natural radioactivity in granites and gneisses of the Opava Mountains (Poland): a comparison between laboratory and in situ measurements

**DOI:** 10.1007/s10967-018-5726-3

**Published:** 2018-02-14

**Authors:** Agnieszka Dżaluk, Dariusz Malczewski, Jerzy Żaba, Maria Dziurowicz

**Affiliations:** Faculty of Earth Sciences, University of Silesia, Będzińska 60, 41-200 Sosnowiec, Poland

**Keywords:** Natural radioactivity, Opava Mountains, Laboratory measurements, In situ measurements

## Abstract

The natural radioactivities of five characteristic igneous rocks of the eastern foreland of the Opava Mountains (Eastern Sudetes, Poland), obtained in the laboratory and under in situ conditions, are presented. The activity concentrations of ^232^Th, ^238^U, and ^40^K were measured using an HPGe gamma-ray spectrometry system. The ranges of the activity concentrations of ^232^Th were 7–71 Bq kg^−1^ in the laboratory and 6–68 Bq kg^−1^ for the in situ measurements. For ^238^U, the ranges of the activity concentrations were 5–52 Bq kg^−1^ in the laboratory and 9–48 Bq kg^−1^ for the in situ measurements, and for ^40^K, the ranges were 520–1560 Bq kg^−1^ in the laboratory and 537–1700 Bq kg^−1^ for the in situ measurements. These determined activity concentrations were compared with the average activity concentrations of the radionuclides in similar types of rocks and with data from the Sudetes available in the literature. No significant differences were found between the in situ and laboratory measurements.

## Introduction

The investigated area includes the Opava Mountains, which are the farthest eastern mountain range in the Eastern Sudetes (SW Poland). These mountains run almost latitudinally along the Polish border with the Czech Republic. To the north, the Opava Mountains lie alongside the Głubczyce Plateau and, in the northwest, they border the Paczkowskie Foothills. Only a small part of these mountains is located in Poland, with the rest situated mostly in the Czech Republic. The highest peak (Příčný vrch, 975 m a.s.l.) is located on the Czech side.

The Opava Mountains are well known for their richness in rocks and minerals. This region has numerous valuable and attractive geological and mining heritage sites connected with gold exploitation. This paper presents the first results from laboratory and in situ measurements of the natural radioactivity of samples and outcrops of granites and gneisses from this region. The aim of these measurements is to compare the results with those obtained in situ and with the activities of ^40^K, ^232^Th, and ^238^U in similar types of rocks.

## Geological settings and locations of sampling points

The Opava Mountains are characterized by a specific tectonic position. They belong to the western part of the Upper Silesia Block, which together with the Brno Block form the Brunovistulicum structure [1]. The processes that created these mountains were several Variscan deformations caused by collisions of the Bohemian Massif and the Brunovistulicum, metamorphic processes, and overthrustings [2]. The Opava Mountains are composed of rocks of different ages and lithologies. There are five structural stages that run longitudinally [1]. From west to east, the East Sudetic nappe pile includes the Velké Vbrno and Keperník nappes resting on the parautochthonous gneisses of the Desná unit and covered by the allochthonous Devonian volcanosedimentary Vrbno group [3]. The Upper Devonian/Lower Carboniferous Andělská-Hora Formation is the oldest sequence of the Variscan flysch formation. It is built of dark phyllites, metagreywackes, and subordinately metaconglomerates [4]. Only the Horní-Benešov Formation, which is of the Lower Carboniferous age, was not transformed. From the west, it contacts the Andělská-Hora Formation and, from the east, it contacts the Moravice Formation. This formation consists of sandstones, mudshales, and conglomerates [1]. The Žulova Massif is the Variscan granitoid intrusion of the Upper Carboniferous age. It occurs within the apophysis at a thickness of approximately 200 m and indicates the existence of acidic volcanism in this area. The Žulova Massif is covered by the gneisses of the Proterozoic Desna Series, and samples of these were collected as well in the present study.

**Fig. 1:**
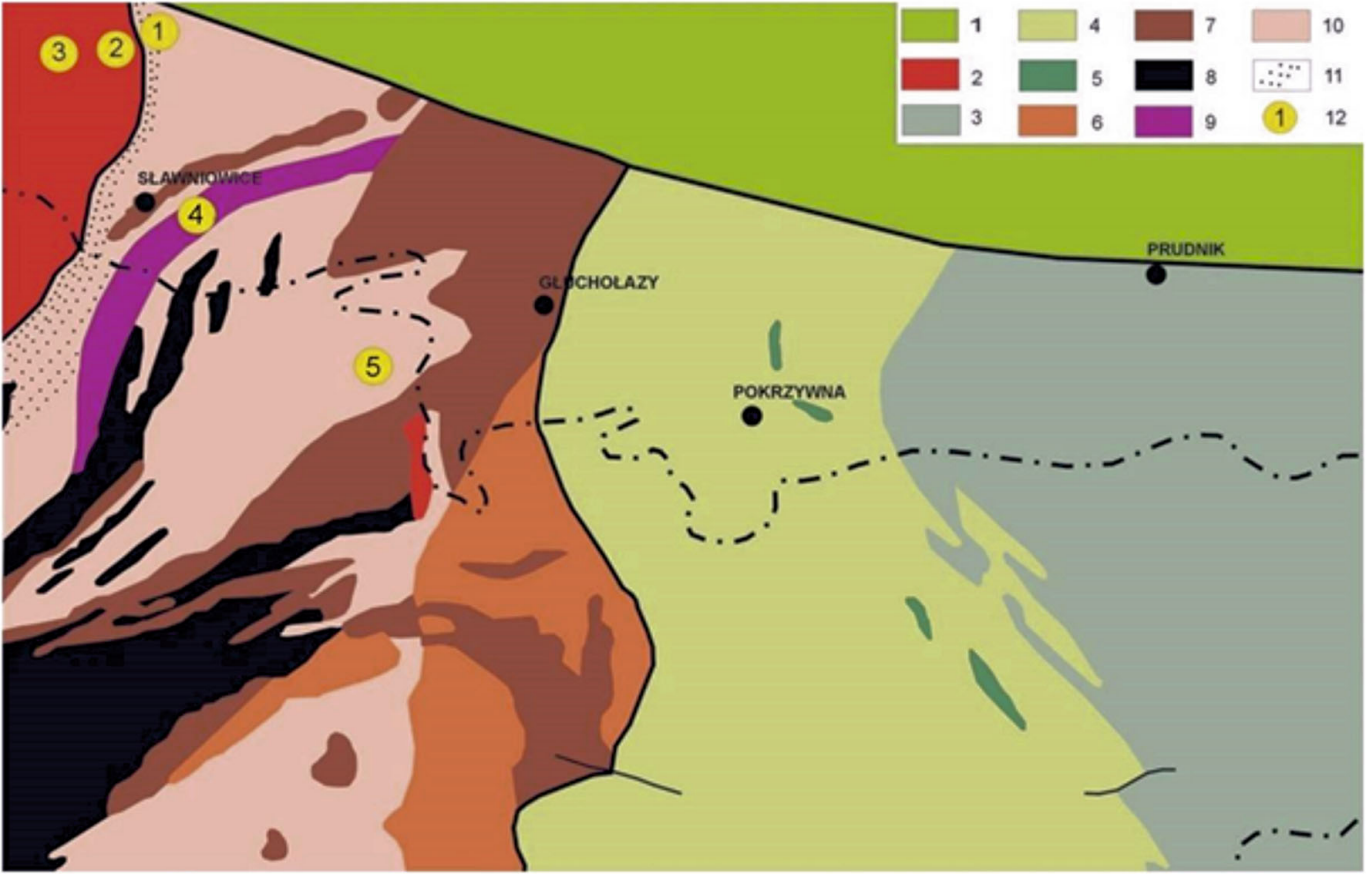
*1* Sandstone and marble (Upper Cretaceous), Žulova Massif (Upper Devonian/Lower Carboniferous, Upper Carboniferous). *2* Granitoid and associated contact phenomena, Horn-Benešov Formation (Lower Carboniferous). *3* Sandstone, mudshale, and conglomerate; Andělská-Hora Formation (Upper Devonian/Lower Carboniferous). *4* Phyllite, metasandstone, crystalline limestone. *5* Greenstone, Vbrna Series (Lower Devonian). *6* Phyllite, graphitic shale, crystalline limestone, and metabasite (Middle Devonian). *7* Quartzite, quartzitic shale, and mica schist; Jesenik Massif. *8* Amphibolite (Lower and Middle Devonian) Desna Series (Proterozoic). *9* Marble *10* gneiss and biotite schist. *11* Contact zone of the Žulova granitoid intrusion. A geological sketch of the Opava Mountains showing locations of in situ measurements and sample collections. *1* Granite, the Kamienna Góra quarry; *2* gneiss, the Kamienna Góra quarry; *3* migmatitic gneiss, the Nadziejów quarry; *4* weathered granite, the Sławniowice quarry; *5* paragneiss, Głuchołazy/Mikulice (after: [1])

The first sample locations in the Kamienna Góra quarry were chosen on natural outcrops of granite (Fig. 1, point 1) and gneiss (Fig. 1, point 2) belonging to the Žulova Massif. The second sample location was in the closed and flooded Nadziejów quarry (Fig. 1, point 3), where migmatitic gneiss occurs. The next rock sample was measured and collected from the Sławniowice quarry. It consisted of medium- and coarse-grained weathered granite of the Upper Carboniferous age (Fig. 1, point 4). The last measured rock was a paragneiss from the Głuchołazy and Mikulice region (Fig. 1, point 5).

## Materials and methods

**Fig. 2:**
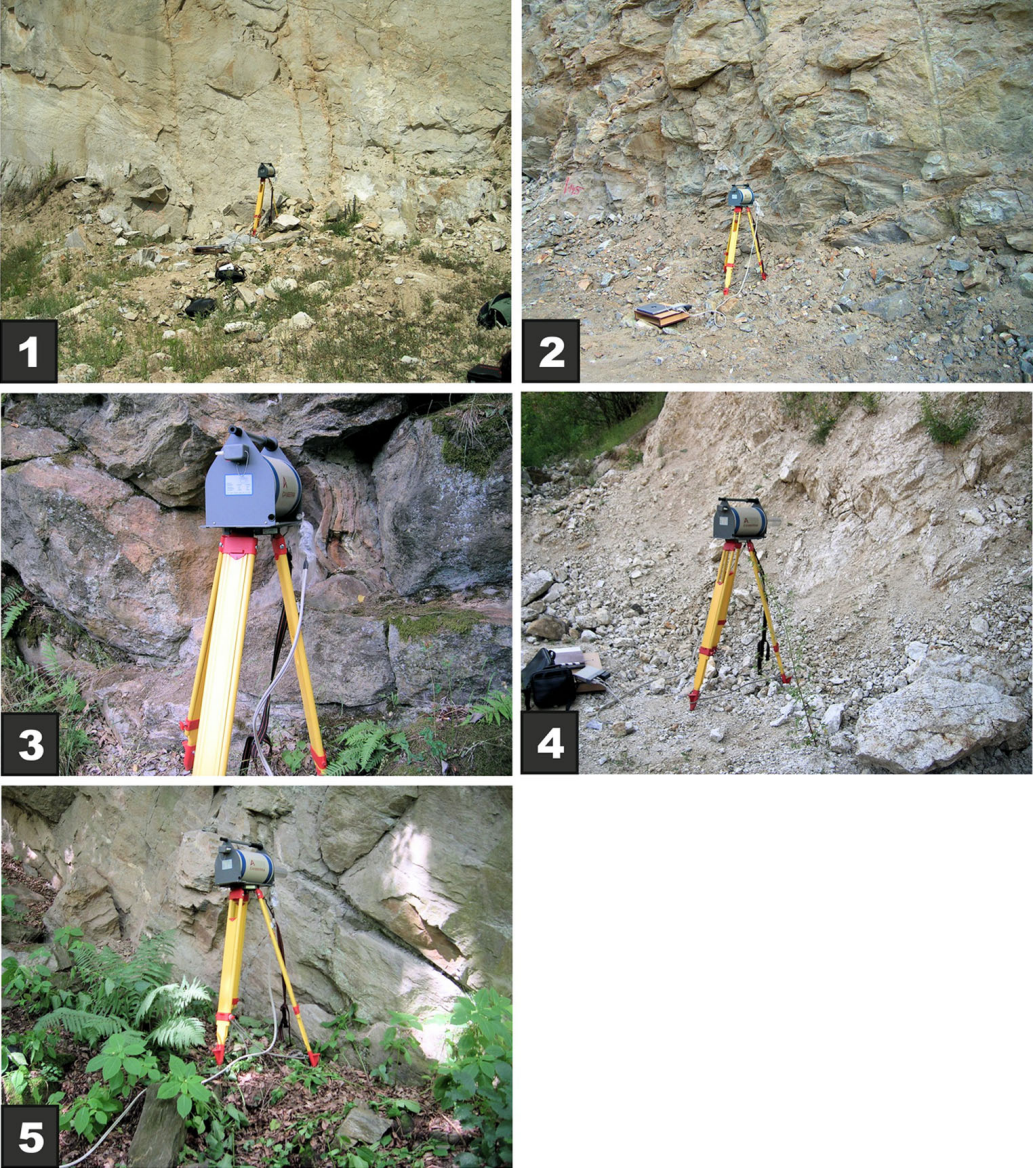
Photos of the site locations and detector configuration for the in situ measurements

The activity concentrations of the naturally occurring radionuclides were measured using the GX3020 gamma-ray spectrometry workstation. The system is based on a high-purity germanium (HPGe) detector with 32% relative efficiency (Fig. 2). The energy resolutions of the detector were 0.8 at 122 keV and 1.7 at 1330 keV.

In the laboratory measurements, the rock samples were crushed and then measured in Marinelli 450 beakers. Each sample was measured for 48 h. The obtained spectra were analyzed using LabSOCS (Laboratory Sourceless Calibration Software) and the Genie 2000 v. 3.4 software package. The ambient background (48 h measurement of an empty Marinelli 450 beaker) was subtracted from each measurement. The spectrometer energy was calibrated using homogeneously dispersed ^241^Am, ^109^Cd, ^139^Ce, ^57^Co, ^60^Co, ^137^Cs, ^113^Sn, ^85^Sr, ^88^Y, and ^203^Hg radioisotopes in a silicone resin [certificate source type Marinelli Beaker Standard Source (MBSS), supplied by the Czech Metrological Institute]. The activities of the examined radionuclides were calculated from the following gamma transitions (energy in keV): ^40^K (1460.8); ^208^Tl (583.1, 860.5, 2614,5); ^212^Pb (238.6, 300.1); ^214^Pb (242, 295.2, and 351.9); ^214^Bi (609.3, 1120.3, and 1764.5); and ^228^Ac (338.32, 911.6, 964.6, and 969.1).

During the in situ measurements, the detector was mounted about 1 m above the ground and 0.2–0.5 m from the outcrop (Fig. 2). These geometries (Fig. 2) capture a majority of the emitted gamma rays from an area with approximately a 2–5 m radius [5]. The calculated average minimum detectable activity (MDA) for ^40^K was 2.7 Bq kg^−1^, whereas the value for ^214^Pb, ^214^Bi, and ^228^Ac was about 1 Bq kg^−1^ [6]. The total duration of a single measurement was 2 h, and in situ object counting software (ISOCS) was used for the efficiency calibration. The consistency of the activities calculated for gamma-ray transitions for a given multiline radionuclide (e.g., ^208^Tl, ^214^Bi, ^214^Pb, ^228^Ac) were checked using a line activity consistency evaluator (LACE) analysis. For all measurements obtained in the laboratory and for the in situ conditions presented here, the activity ratios for the multiline radionuclides were close to unity [7].

Both the LabSOCS and ISOCS procedures require the use of the Geometry Composer in Genie 2000 (v.3). The Geometry Composer parameters that must be specified include the Marinelli beaker and outcrop dimensions, the rock densities and chemical compositions both for laboratory and in situ measurements, and the distance between the rock outcrops and the detector for in situ measurements. The final output from the LabSOCS/ISOCS software package is the full-energy efficiency for a given source-detector geometry. The obtained agreement between model and measurement is within 5% for point-like sources and within 10–15% for extended sources [8].

## Results and discussion

### Laboratory measurements

**Fig. 3:**
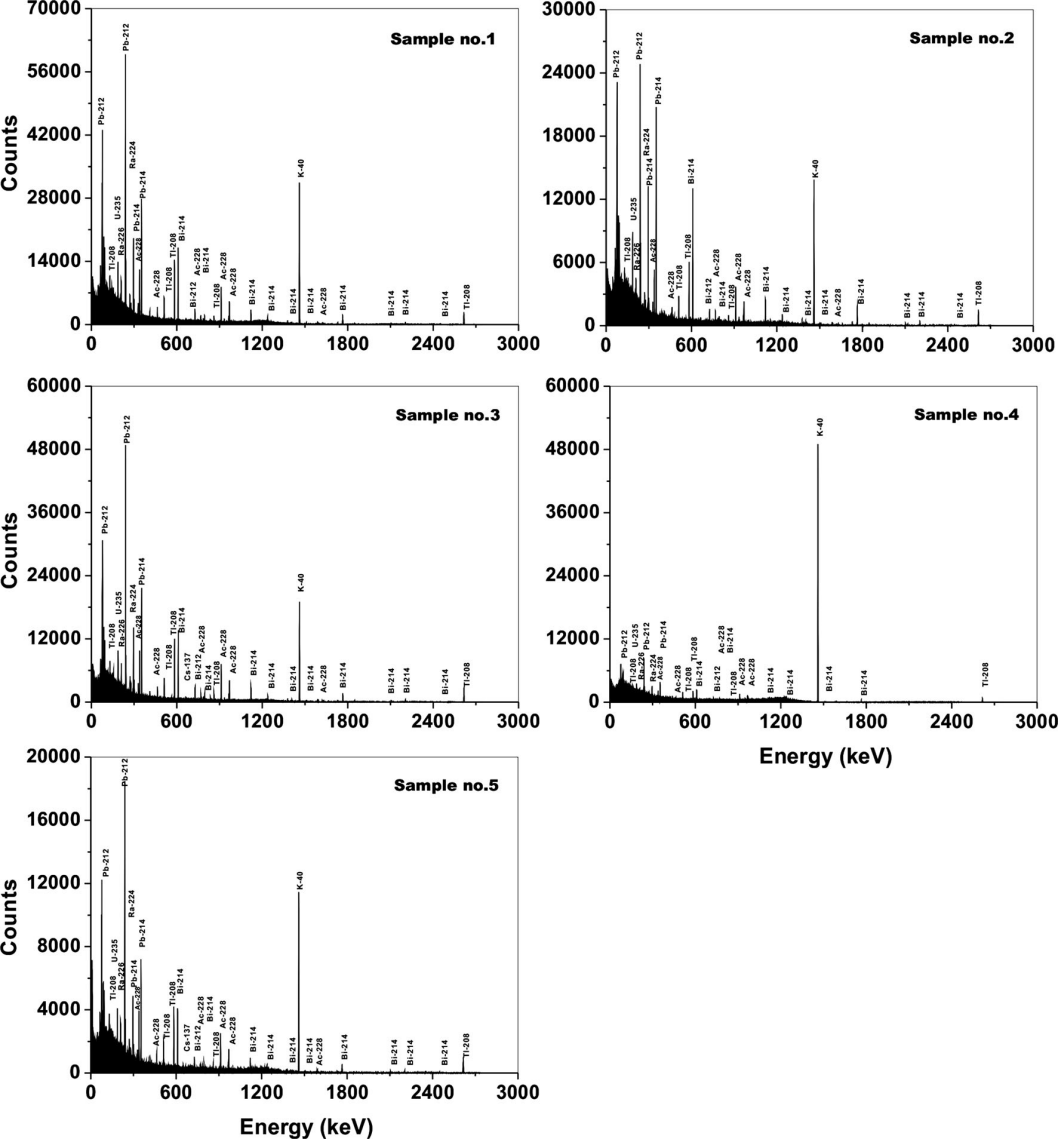
Gamma-ray spectra for all samples. The characteristic γ-ray emitters are marked above the corresponding peaks

The results of the gamma-ray activities of the ^40^K, ^232^Th, and ^238^U series are provided in Table 1. The gamma-ray spectra are shown in Fig. 3.

**Table 1:** Laboratory measured activity concentrations of primordial radionuclides in the examined rock samples. Uncertainties are quoted as 1σ

Nuclide	Activity (Bq kg^−1^)				
	Sample no				
	1 Granite	2 Gneiss	3 Migmatic gneiss	4 Weathered granite	5 Paragneiss
Non series					
^40^K	942 ± 17	695 ± 15	778 ± 17	1560 ± 34	520 ± 11
^232^Th series					
^208^Tl^a^	18.4 ± 0.6	17.7 ± 0.6	25.5 ± 0.9	2.4 ± 0.3	8.7 ± 0.4
^212^Pb	55.7 ± 1.2	52.9 ± 1.2	71.6 ± 1.6	7.2 ± 0.3	26.3 ± 0.6
^228^Ac	53.4 ± 1.5	51.1 ± 1.5	70.6 ± 2.0	7.2 ± 0.5	25.1 ± 0.9
^238^U series					
^214^Pb	44.4 ± 1.2	43.0 ± 1.3	52.6 ± 1.6	5.8 ± 0.3	16.6 ± 0.6
^214^Bi	43.2 ± 1.1	42.1 ± 1.1	52.1 ± 1.4	5.2 ± 0.5	15.8 ± 0.7
^226^Ra^b^	43.8 ± 1.6	42.6 ± 1.7	52.4 ± 2.1	5.5 ± 0.6	16.2 ± 0.9

^a^Branching ratio 36%

^b^Based on ^214^Pb and ^214^Bi activities. Uncertainties were calculated as: Δ^226^Ra = ((Δ^214^Pb)^2^ + (Δ^214^Bi)^2^)^1/2^

### ^40^K

**Fig. 4:**
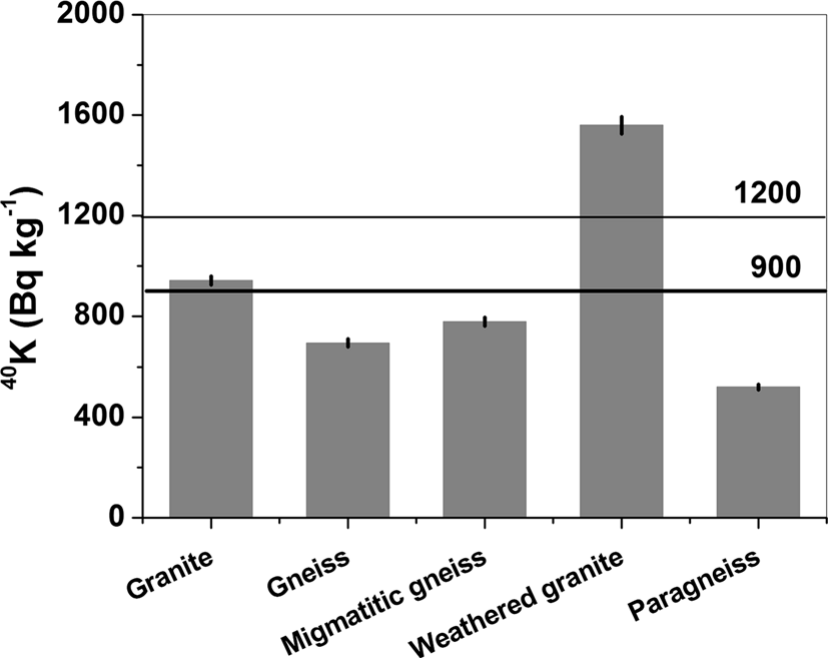
Activity concentrations of ^40^K. Thick solid line: average ^40^K value from all samples. Thin solid line: average ^40^K activity in typical granite

As is shown in Table 1, the lowest activity of ^40^K was recorded in sample 5 (paragneiss, 520 Bq kg^−1^) from Głuchołazy, while the maximum activity was observed in sample 4 (weathered granite, 1560 Bq kg^−1^). The next highest value, 942 Bq kg^−1^, occurred in sample 1 (granite). The activity of ^40^K in sample 4 was nearly twice as high as the activity concentration in the non-weathered granite from the Kamienna Góra quarry. This is caused by the large amount of potassium feldspar in the weathered granite from Sławniowice [9]. Intermediate values were noted in sample 2 (gneiss, 645 Bq kg^−1^) and sample 3 (migmatic gneiss, 778 Bq kg^−1^). The activity of ^40^K averaged over all the samples was 900 Bq kg^−1^, which is lower than the averaged activity, about 1200 Bq kg^−1^, that was reported for typical granite [10, 11] (Fig. 4). This value was only exceeded in sample 4 (weathered granite). Generally, except for the weathered granite, the activity of ^40^K in the investigated rocks was lower than that measured in similar rocks of the Izera Block (Western Sudetes) [12, 13]. Granite from Kamienna Góra (sample 1) is also characterized by lower ^40^K activity than in granites from the Bohemian Massif [14], Brazil [15, 16], Caucasus [17], Egypt [18], Yemen [19] and Chinese commercial granites [20]. The rock samples from the Kestanbol granitic plutons from the Ezine region [21] and from the Kaymaz and Sivrihisar plutons [22] located in Turkey show the higher average activity concentrations as well. On the other hand, exceptionally low ^40^K activity (261 Bq kg^−1^) was recorded in granite from Saudi Arabia [23].

^40^K activities that were similar to these observed in sample 2 (gneiss) and sample 3 (migmatite gneiss) were reported for the gneisses of the Bohemian Massif [14] and Koprubasi (Turkey) [24]. The recorded ^40^K activity was definitely higher in the gneisses from Brazil [15, 16]. Paragneiss from Głuchołazy (sample 5) is characterized by lower ^40^K activity than paragneisses from the Izera Block [13] and South Bohemia [14].

### ^232^Th series (^208^Tl, ^212^Pb, ^228^Ac)

**Fig. 5:**
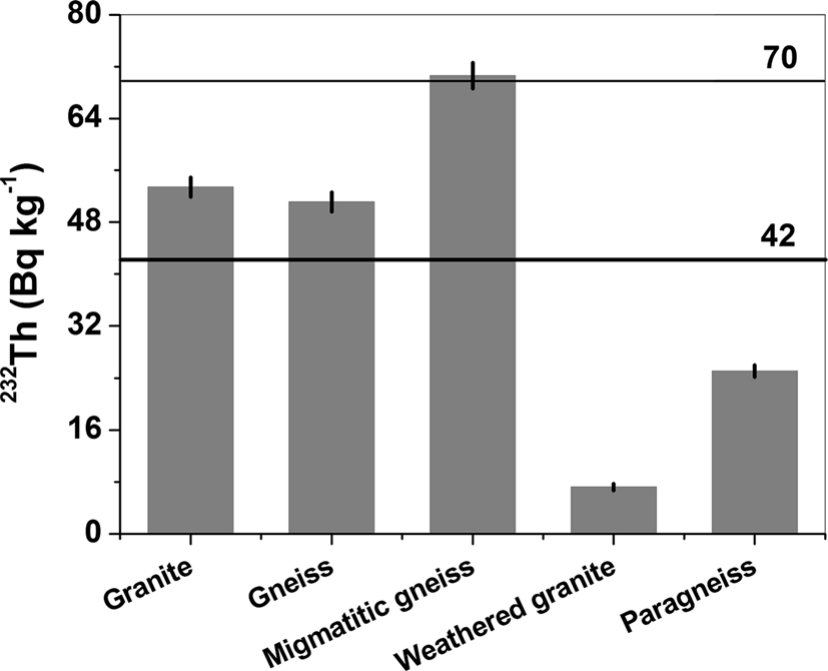
Activity concentrations of ^232^Th. Thick solid line: average ^232^Th value from all samples. Thin solid line: average ^232^Th activity in typical granite

The data presented in Table 1 show that all of the rock samples achieve radioactive equilibrium between the progenies in the ^232^Th series. Since ^228^Ac is the second radionuclide in the thorium series, the activity concentration of ^232^Th can be determined based on the ^228^Ac activity. The highest activity concentration of ^232^Th was measured in migmatic gneiss from Nadziejów (sample 3, 71 Bq kg^−1^), whereas the lowest activity was found in weathered granite from the Sławniowice quarry (sample 4, 7 Bq kg^−1^). Values of 51 and 25 Bq kg^−1^ were measured in gneiss from Kamienna Góra (sample 2) and paragneiss from Głuchołazy (sample 5), respectively. The exceptionally low activity of ^232^Th in sample 4 results from a lack of dark minerals caused by the weathering process. The ^232^Th activity averaged over all samples was 42 Bq kg^−1^ (Fig. 5). An arithmetic mean of the ^232^Th activity, excluding sample 4, is 50 Bq kg^−1^. Similar to the ^40^K activity, both averages are below the mean of 70 Bq kg^−1^ reported for ^232^Th in typical granite [10]. This value was only achieved in sample 3 (migmatic gneiss) (Fig. 5; Table 1). The average activity of 42 Bq kg^−1^ is the same as that observed in granites and gneisses from the Izera Block obtained using an in situ field method [12, 13]. Similar results, obtained in granite from Kamienna Góra quarry (sample 1, 53 Bq kg^−1^), were reported in the region of the Great Caucasus [17], Spain [25], and Italy [23], whereas the activity of thorium observed in Brazilian [15, 16] and Chinese granites [20] was significantly higher. Low ^232^Th activities of 8 and 13 Bq kg^−1^ were found for granites located in Saudi Arabia [23] and cista type granite in the Bohemian Massif [14], respectively. These activities are comparable with the value in weathered granite from Sławniowice quarry (sample 4). The activity concentration of ^232^Th in ortho-gneiss from Kamienna Góra quarry (sample 2, 51 Bqkg^−1^) is comparable with data reported in the literature [14, 24]. Exceptionally high activities that even exceeded 200–300 Bq kg^−1^ were reported in gneisses from Yemen [19], Brazil [15] and in granites from Turkey [21, 22]. The migmatic gneiss from Nadziejów (sample 3) gave the highest ^232^Th activity of 71 Bq kg^−1^. Similar values, ranging from 78 to 81 Bq kg^−1^, were observed in migmatic gneisses from Brazil [15], whereas migmatic gneiss from South Bohemia was characterized by a significantly lower thorium activity of about 24 Bq kg^−1^ [14].

### ^238^U series (^214^Pb, ^214^Bi, ^226^Ra)

The activity concentrations of ^238^U were estimated assuming radioactive equilibrium in the ^238^U → ^226^Ra → ^222^Rn → ^214^Pb → ^214^Bi decay chain, which occurs in the vast majority of minerals and rocks [26, 27]. The ^238^U activity concentration was based on the ^226^Ra activity concentration, and the ^226^Ra activity concentration was calculated as the arithmetic mean of the activities of the ^214^Pb and ^214^Bi isotopes. The results, which are summarized in Table 1, show that the activity concentrations of ^238^U varied from ~ 6 Bq kg^−1^ (weathered granite, sample 4) to 52 Bq kg^−1^ (migmatic gneiss, sample 3). Granite (sample 1) and gneiss (sample 2) had activities of 44 and 43 Bq kg^−1^, respectively. A relatively low activity for ^238^U was noted in paragneiss (sample 5, 16 Bq kg^−1^).

**Fig. 6:**
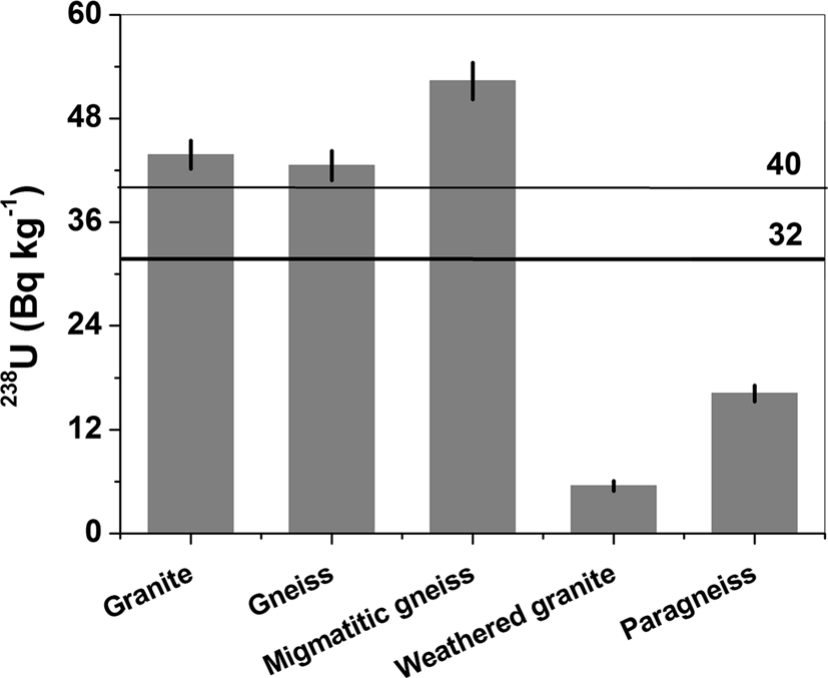
Activity concentrations of ^238^U. Thick solid line: average ^238^U value from all samples. Thin solid line: average ^238^U activity in typical granite

Figure 6 shows an average ^238^U activity of 32 Bq kg^−1^. Excluding sample 4, the average is 39 Bq kg^−1^. Both of the averages are close to those reported for typical granite, 40 Bq kg^−1^ [10, 11]. However, these averaged values are lower than the arithmetic mean of 57 Bq kg^−1^ calculated from 48 samples of igneous rocks from the Sudetes (without the Opava Mountains) [28] and the arithmetic mean of 58 Bq kg^−1^ calculated for igneous rocks of the Izera Mountains only [12, 13]. These values are also significantly lower than those measured in Turkey [21, 22], where, in the Kaymaz pluton, the average activity concentration of uranium amounted to more than 300 Bq kg^−1^). The activity of ^238^U for granite from Kamienna Góra (sample 1) was reported to be similar to granites from Mexico [29] and Yemen [19]. The activities measured in the region of the Great Caucasus [17] and Saudi Arabia [23] were lower, but still significantly higher than that in weathered granite from the Sławniowice quarry (sample 4). The uranium activities recorded in granites from the Bohemian Massif [14], Egypt [18], and Brazil [16] were definitely higher (118–204 Bq kg^−1^) than that noted in sample 1. The activity concentration of ^238^U in gneiss from Kamienna Góra (sample 2, 43 Bq kg^−1^) measured in the Opava Mountains is nearly the same as the average reported in gneisses from the Bohemian Massif, ~ 41 Bq kg^−1^. Slightly higher activities have been reported in other localities, e.g., in Brazil [16], Yemen [19] and China [20]. Migmatic gneiss (sample 3) showed twice the uranium activity than those noted in Brazil [15] and in the Bohemian Massif [14]. The measured uranium activity in paragneisses (sample 5, 16 Bq kg^−1^) is lower than that reported for paragneisses from the Bohemian Massif (21–42 Bq kg^−1^) [14, 29].

### Estimates of K (%), ^232^Th (ppm), and ^238^U (ppm)

Based on the activities given in Table 1, the concentrations of K (%), ^232^Th (ppm), and ^238^U (ppm) were calculated (Table 2). The concentration of K ranged from 1.7 wt% (sample 5, paragneiss) to 5.1 wt% (sample 4, weathered granite). The concentration of Th varied from 1.8 ppm in sample 4 (weathered granite) to 17.3 ppm in sample 3 (migmatic gneiss). As with the Th concentration, the lowest U concentration, 0.4 ppm, occurred in sample 4 and the highest concentration, 4.2 ppm, was found in sample 3.

**Table 2:** Concentrations of K (%), ^232^Th (ppm) and ^238^U (ppm)

Nuclide	Activity (Bq kg^−1^)				
	Sample no.				
	1 Granite	2 Gneiss	3 Migmatic gneiss	4 Weathered granite	5 Paragneiss
K (%)^a^	3.1 ± 0.1	2.3 ± 0.05	2.6 ± 0.1	5.1 ± 0.1	1.7 ± 0.04
^232^Th (ppm)	13.1 ± 0.4	12.6 ± 0.4	17.3 ± 0.5	1.8 ± 0.1	6.2 ± 0.2
^238^U (ppm)	3.5 ± 0.1	3.4 ± 0.1	4.2 ± 0.2	0.4 ± 0.05	1.3 ± 0.07

^a^The following conversion factors were used: K (%) × 303.16 = ^40^K (Bq kg^−1^); ^232^Th (ppm) × 4.07 = ^232^Th (Bq kg^−1^); and ^238^U (ppm) × 12.36 = ^238^U (Bq kg^−1^)

**Fig. 7:**
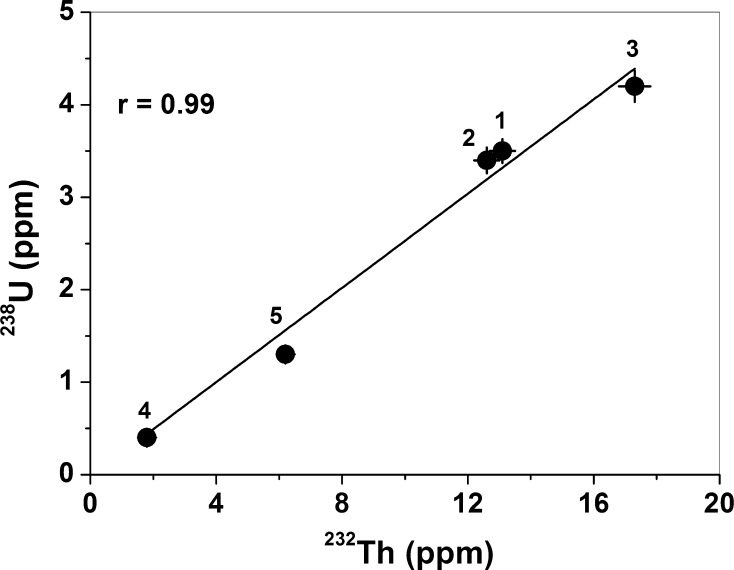
Correlation between ^232^Th (ppm) and ^238^U (ppm). The solid red lines represent the linear fit, ^238^U (ppm) = 0.26 × ^232^Th (ppm). The correlation coefficient is *r* = 0.99

As can be seen in Fig. 7, there is a strong positive correlation between the ^238^U and ^232^Th concentrations in the investigated rocks, with a correlation coefficient of 0.99. This strong correlation indicates that the thorium and uranium concentrations diminished proportionally during the weathering process in granite from the Sławniowice quarry (sample 4). Since the Th and U concentrations in the weathered granite are exceptionally low, there are only negative correlations between K and Th and K and U for all of the investigated rocks. Excluding sample 4, the correlation coefficients of K–Th and K–U are 0.74 and 0.81, respectively.

### Comparison between in situ and laboratory measurements

Preliminary results and a discussion of in situ measurements of four of these rocks were presented in previous work by Dżaluk et al. [30]. After additional measurements, the previous results were slightly modified and the results of activities in paragneiss from Głuchołazy (location 5) were added. The resulting in situ measurements in the granites and gneisses of the Opava Mountains are listed in Table 3.

**Table 3:** In situ measured activity concentrations of primordial radionuclides at specified locations. Uncertainties are quoted as 1σ

Nuclide	Activity (Bq kg^−1^)				
	Sample no.				
	1 Granite	2 Gneiss	3 Migmatic gneiss	4 Weathered granite	5 Paragneiss
Non series					
^40^K	932 ± 21	700 ± 15	806 ± 17	1700 ± 38	537 ± 12
^232^Th series					
^208^Tl^a^	20.1 ± 0.8	17.1 ± 0.7	25.9 ± 1.1	2.5 ± 0.2	9.5 ± 0.4
^212^Pb	55.9 ± 2.3	47.0 ± 2.2	70.7 ± 2.6	6.4 ± 0.7	26.9 ± 1.1
^228^Ac	56.0 ± 1.7	46.6 ± 1.7	70.6 ± 2.0	7.0 ± 0.6	26.2 ± 1.2
^238^U series					
^214^Pb	35.9 ± 1.6	46.4 ± 1.5	47.9 ± 1.9	8.6 ± 0.2	18.8 ± 0.9
^214^Bi	35.3 ± 1.7	44.7 ± 1.6	48.4 ± 1.7	8.8 ± 0.3	18.7 ± 0.4
^226^Ra^b^	35.6 ± 2.3	45.6 ± 2.2	48.2 ± 2.5	8.7 ± 0.4	18.8 ± 1.0

^a^Branching ratio 36%

^b^Based on ^214^Pb and ^214^Bi activities. Uncertainties were calculated as: Δ^226^Ra = ((Δ^214^Pb)^2^ + (Δ^214^Bi)^2^)^1/2^

**Fig. 8:**
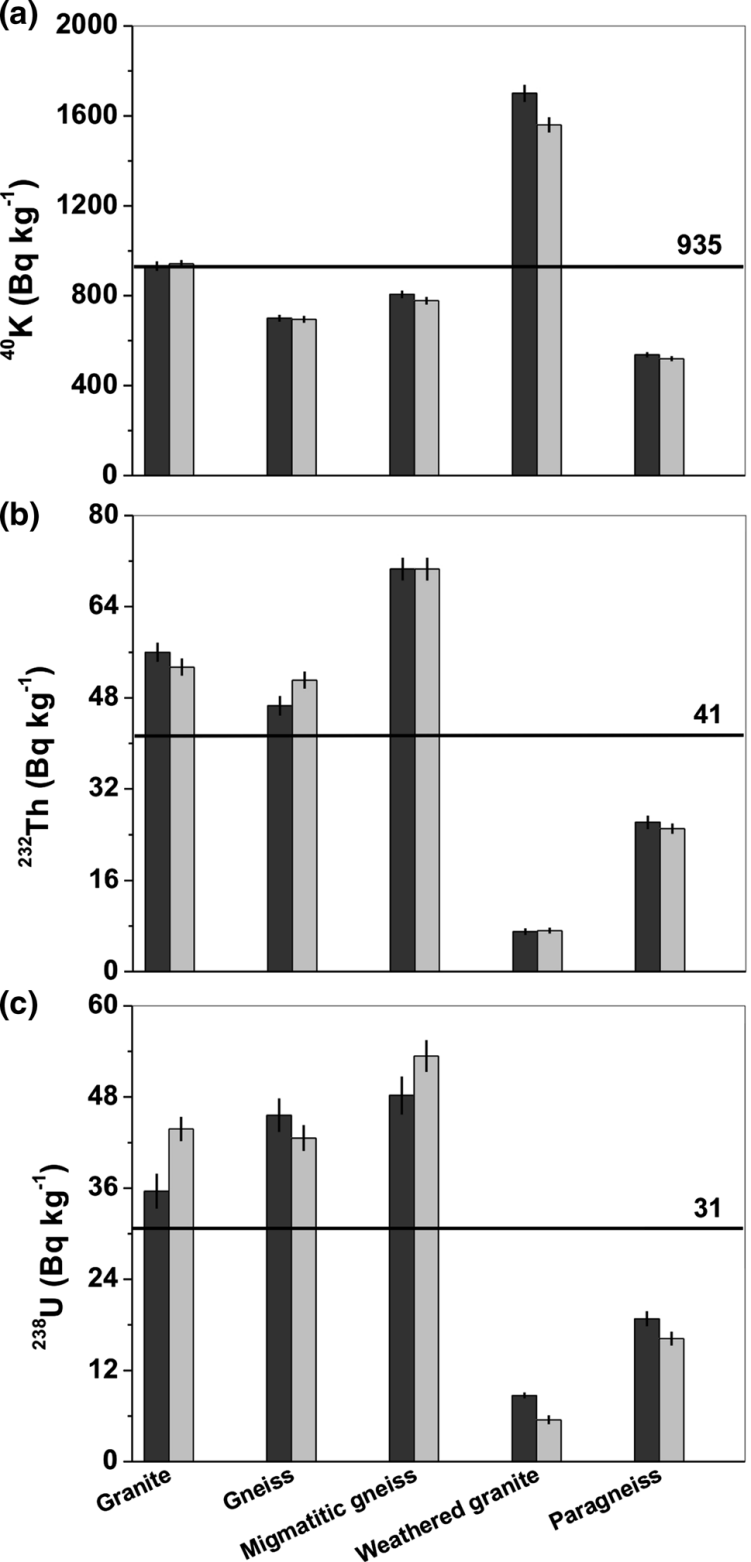
Comparison between in situ (black bars) and laboratory (gray bars) measurements: **a** ^40^K, **b** ^232^Th, **c** ^238^U. The horizontal line represents the in situ averaged value calculated over all the rocks

As seen in Fig. 8a–c, the results obtained in situ and in laboratory conditions agree well. The in situ activities of ^40^K, ^232^Th, and ^238^U, averaged over all locations, were 935, 41, and 31 Bq kg^−1^, respectively. The in situ averages for ^232^Th and ^238^U are the same, within the level of uncertainty, as these obtained in laboratory measurements. A noticeable difference in the ^40^K activity of 140 Bq kg^−1^ between the in situ and laboratory measurements only occurred for weathered granite (location and sample 4) (Fig. 8a). For other locations, the calculated average of the absolute differences between the in situ and laboratory measurements |^40^K_in situ_—^40^K_lab_| was 15 Bq kg^−1^. This value is close to the individual uncertainties presented in Tables 1 and 3. Figure 8b shows that the smallest differences between the in situ and laboratory results were observed for the ^232^Th activities. An average from the absolute differences of |^232^Th_in situ_—^232^Th_lab_| was 1.7 Bq kg^−1^. Similar to the results for potassium, this average is practically the same as the individual uncertainties given in Tables 1 and 3. For gneiss (location and sample 2), the ^232^Th activity obtained in the laboratory exceeded the activity from the in situ measurement by 4.5 Bq kg^−1^. For ^238^U, the largest differences between the laboratory and in situ measurements were observed in granite (location and sample 1) and migmatic gneiss (location and sample 3); these differences were 8.2 and 4.2 Bq kg^−1^, respectively (Fig. 8c). An average of the absolute differences of |^238^U_in situ_—^238^U_lab_| for all the locations and samples was 4 Bq kg^−1^, and when excluding granite, the average was 3.2 Bq kg^−1^.

## Conclusions

The results of laboratory and in situ gamma-ray measurements in granites and gneisses in the Opava Mountains are consistent. The highest ^40^K activity concentration was observed in the granites, whereas the highest ^232^Th and ^238^U activities were measured in migmatic gneiss. The exceptionally low ^232^Th and ^238^U activities were observed in weathered granite. The activity concentrations of ^40^K, ^232^Th, and ^238^U averaged over all rock samples were 900, 41, and 31 Bq kg^−1^, respectively. In the investigated rocks, the concentrations of ^232^Th (ppm) and ^238^U (ppm) showed a strong positive correlation.
